# Parasitic Recognition Behavior of *Telenomus remus* Nixon, an Important Egg Parasitoid of *Spodoptera frugiperda* (J. E. Smith)

**DOI:** 10.3390/insects17010093

**Published:** 2026-01-14

**Authors:** Xiaolong Ma, Yujie Luo, Qiufen Zhao, Ruohan Zhang, Haiyan Lin, Jian Huang, Zhuhong Wang

**Affiliations:** State Key Laboratory of Agricultural and Forestry Biosecurity, College of Plant Protection, Fujian Agriculture and Forestry University, Fuzhou 350002, China; mxl19993449499@163.com (X.M.); 13705952507@163.com (Y.L.); 18649905806@163.com (Q.Z.); zrh8109@163.com (R.Z.); lh2637025761@163.com (H.L.); jhuang1234@126.com (J.H.)

**Keywords:** *Telenomus remus* Nixon, parasitic recognition behavior, oviposition experience, temperature, time intervals

## Abstract

*T. remus* is an important egg parasitoid of *S. frugiperda*, serving as a significant role in its biological control. This study systematically examined the host discrimination behavior of *T. remus*. The parasitic process comprises several distinct behavioral stages: host searching, antennal tapping and examination, ovipositor probing, “8”-shaped marking, and grooming. Following successful oviposition, females perform a characteristic “8”-shaped marking on the host egg surface with their ovipositor, which deters conspecific females from parasitizing the same host. *T. remus* exhibited a pronounced ability to discriminate parasitized hosts, utilizing both antennae and ovipositor to avoid superparasitism. As host density increased, the searching time of *T. remus* decreased while the parasitism rate increased, eventually stabilizing. Parasitic discrimination was significantly influenced by oviposition experience: experienced females effectively recognized marked host eggs across a temperature range of 16 to 36 °C and time intervals of 0 to 12 h post oviposition. In contrast, naive females exhibited discrimination ability only at lower temperature (16 °C) and immediately following oviposition (0 h). These findings deepen the understanding of the behavioral ecology of *T. remus* and provide a crucial theoretical basis for its efficient application in the biological control of *S. frugiperda*.

## Simple Summary

The fall armyworm, *Spodoptera frugiperda*, is a major pest of maize. Since its invasion of China in 2019, it has rapidly established itself and spread to most regions of the country. Its polyphagous feeding habits, high feeding capacity, rapid development cycle, robust reproductive potential, resistance to pesticides, and migratory tendencies pose significant challenges for its control. Utilizing natural enemies for biological control is therefore an essential strategy. The parasitoid, *Telenomus remus*, serves as an important egg parasitoid targeting various lepidopteran pests, and has demonstrated substantial efficacy against *S. frugiperda*. This study examined the host discrimination and marking behavior of *T. remus*, specifically assessing its ability to identify host eggs marked by itself or conspecific individuals under different temperature conditions and oviposition intervals. The results contribute to a deeper understanding of the parasitic behavioral traits of *T. remus* and offer an essential scientific foundation for its mass rearing and application in biological control programs.

## 1. Introduction

To ensure sufficient nutrition for their progeny, female parasitoids generally avoid ovipositing on hosts that have already been parasitized, thereby reducing competition among offspring for host resources [1]. This ability is known as host discrimination [2]. Host discrimination can be further delineated into intraspecific and interspecific recognition, with the former encompassing self-discrimination and conspecific discrimination [3]. Self-discrimination pertains to the female parasitoid’s ability to identify hosts they have previously parasitized, whereas conspecific discrimination involves distinguishing hosts parasitized by other females of the same species [3,4]. Parasitoids predominantly utilize sensory organs such as antennae and ovipositors to facilitate host discrimination. The sensilla located on the antennae are chiefly responsible for detecting external cues on the host surface, while those on the ovipositor assess the internal condition of the host [5].

A key mechanism underlying host discrimination is the use of marking pheromones. During oviposition, parasitoids frequently deposit marking substances on the host surface, which can be detected by subsequent females to avoid superparasitism [6,7,8]. More than 200 species of female wasps from Ichneumonoidea, Chalcidoidea, Proctotrupoidea, Bethyloidea, and Cynipoidea have been reported to use host markers to identify already parasitized hosts [9]. For example, females of *Aprostocetus prolixus* LaSalle & Huang apply a marking secretion to the oviposition slit after egg-laying, which deters subsequent parasitoids from parasitizing the same host [10]. These host-marking pheromones consist mainly of saturated and unsaturated hydrocarbons [11], and most parasitoids depend on them for recognizing parasitized hosts [11,12].

The efficiency of host recognition and superparasitism avoidance is influenced by multiple factors. These include the time elapsed since the host was last parasitized, the developmental stage of parasitoid eggs within the host, the quantity of eggs previously deposited, host density, the duration of activity of host-marking compounds, and the interval between oviposition events due to a shortage of suitable hosts [3,13,14,15,16]. Typically, upon detecting a marked or parasitized host, a parasitoid will abandon it and search for unparasitized ones, thereby enhancing biological control efficacy. However, the ability to recognize these marks declines over time [17,18,19]. There are two main mechanisms proposed to explain this phenomenon:

The first involves the chemical properties of the pheromone itself. One hypothesis suggests that the marking pheromone comprises two functional components: a short-lived, highly active component that primarily inhibits the marking female from re-ovipositing in the same host, and a longer-lasting, less active component that mainly deters conspecifics. Over time, the short-lived component degrades, reducing its self-recognition effect, while the more stable, long-lasting component continues to provide a degree of within-species recognition [20]. Environmental factors such as temperature can accelerate the volatilization and degradation of these pheromone molecules, shortening the effective recognition window [21]. For instance, the host-marking pheromone deposited by *T. podisi* Ashmead and *T. euschisti* Ashmead on stink bug eggs remains effective for recognition only within one hour after secretion [22]. Thus, factors such as temperature and time intervals may interfere with the parasitoid’s ability to distinguish between its own marks and those of others.

The second involves the cognitive processes of parasitoids—learning and memory. The parasitoids retain a transient memory of having parasitized a host, but this memory weakens and ultimately disappears over time [23]. In fact, the learning and memory behavior also plays a crucial regulatory role in host recognition. For example, *Trichogramma ostriniae* Pang et Chen exhibit both learning and memory capabilities and can acquire host-marking cues to enhance host-searching efficiency and modify its oviposition strategy [24]; *Anastatus japonicus* Ashmead shows significantly stronger attraction to volatiles emitted by litchi stink bugs after training with β-caryophyllene [25]; *Asobara japonica* Belokobylskij improves its recognition ability through repeated parasitism experiences [26]; *Sclerodermus pupariae* Yang et Yao demonstrates increased search efficiency following initial host contact, indicating that learning reinforces its host preference [27]. These species, *T. ostriniae*, *A. japonicus*, *A. japonica*, and *S. pupariae* are all advantageous for large-scale biological control. Through host recognition, continuous learning allows parasitoids to accumulate discriminatory experience, optimize foraging behavior, and improve host selection efficiency, thereby minimizing time wasted on already parasitized hosts.

Many parasitoids in the genera *Telenomus* and *Trissolcus* possess host recognition abilities. However, while most *Trissolcus* species employ a uniform marking method, *Telenomus* species exhibit diverse recognition-marking strategies (Table 1). In China, *T. remus* Nixon serves as a key parasitoid of *Spodoptera frugiperda* (J. E. Smith) and plays a significant role in its biological control. Nonetheless, as a monophagous parasitoid, superparasitism can still occur under mass-rearing conditions [3]. Comprehensive studies on the host discrimination behavior of *T. remus* remain scarce. Understanding this behavior is essential for improving its field deployment and enhancing its host selection efficiency under practical conditions.

Therefore, this study takes *T. remus* as the object, through observing its marking behavior on the surface of the host, dissecting the host to confirm the internal parasitic state, and comparing experiments under different temperature conditions and time intervals, in order to explore the following: (1) whether and how the marking of the host egg affects the recognition behavior of the parasitoid by volatilization or degradation; (2) a systematic evaluation of the self- and conspecific recognition behavior of *T. remus* toward marked host eggs. The findings are expected to provide scientific guidance for optimizing the field application strategy of *T. remus*.

## 2. Materials and Methods

### 2.1. Insect Sources

*S. frugiperda* larvae were initially collected from a maize field located in Shoushan, Fuzhou City, Fujian Province. A laboratory colony has since been established and maintained for over 10 generations under controlled conditions of 26 ± 1 °C, relative humidity of 70 ± 5%, a photoperiod of 16L:8D, and a light intensity of 1000 lux.

*T. remus* were also collected from the same site and have been continuously reared in the laboratory using *S. frugiperda* eggs as hosts. The rearing conditions were maintained at 26 ± 1 °C, relative humidity of 70 ± 10%, a photoperiod of 14L:10D and a light intensity of 1000 lux. Insects from both colonies were reared for multiple generations under these controlled conditions, and specimens were reserved for subsequent experiments.

### 2.2. Experimental Apparatus

The following instruments and equipment were used in this study: an artificial climate incubator (Model MRX-350B-LED with top-mounted LEDs, manufactured by Ningbo Prante Instrument Co., Ltd., Ningbo, China); a NikonNi microscope equipped with differential interference contrast (DIC) optics, coupled with a NikonDS-Ri2 camera and operated using NIS-Elements D v4.4 software (Nikon Corporation, Shanghai, China); a Nikon SMZ745 stereomicroscope (Nikon Corporation, Shanghai, China); an Haier DW-86L338 ultra-low temperature freezer (Haier Company, Fuzhou, China); handmade dissection needles; and multiple finger tubes (φ = 0.5 cm, h = 3 cm).

### 2.3. Test Methods

#### 2.3.1. Observation of Parasitization Behavior in *T. remus*

The parasitization behavior of *T. remus* was observed by placing a newly emerged (≤3 h old), healthy female individual together with a fresh *S. frugiperda* egg mass (≤24 h old) inside a finger tube. A strip of filter paper soaked in a 20% honey solution was provided within the tube as a nutritional source. The parasitic behavior was continuously monitored for a duration of 30 min using a stereomicroscope, and photographic documentation was conducted to record the observed behaviors. This procedure was replicated five times.

#### 2.3.2. Parasitism Efficiency of an Individual *T. remus* Female on Host Eggs at Different Densities

Fresh egg masses of *S. frugiperda* (≤24 h old) were prepared at densities of 10, 16, 20, 30, 40, and 50 eggs per mass. Newly emerged, healthy female *T. remus* individuals were collected for the study. Each experimental unit consisted of a single female introduced into a finger tube containing one host egg mass of a designated density. The number of parasitized eggs, identified by characteristic marking behavior, was recorded at two time intervals, 0–10 min and 0–30 min, to determine the parasitism rate. The experiment was conducted at room temperature and replicated five times.

#### 2.3.3. Parasitism Rate of an Individual *T. remus* Female in Relation to Host Exposure Time

Based on prior findings, a host egg density of 30 eggs per mass was selected as optimal for parasitization and observation. Fresh *S. frugiperda* egg masses (30 eggs/mass, ≤24 h old) were exposed to single, newly emerged, healthy females of *T. remus* in finger tubes. These tubes were maintained in an artificial climate chamber set at 26 ± 1 °C, 70 ± 5% relative humidity, with a 14L:10D photoperiod, and a light intensity of 1000 lux. The females were allowed to parasitize the host eggs for designated exposure periods of 1, 1.25, 1.5, 1.75, 2, 2.25, or 2.5 h, after which they were removed. Parasitism was confirmed if, within three days, the host eggs remained plump, turned progressively black, and failed to hatch *S. frugiperda* larvae. The parasitism rate was calculated and recorded accordingly. The experiment was replicated 10 times for each exposure duration.

#### 2.3.4. Dissection-Based Examination of Host Discrimination by *T. remus* Toward Previously Parasitized Host Eggs

Fresh *S. frugiperda* egg masses (30 eggs/mass, ≤24 h old)were parasitized by *T. remus* females one hour prior to the experiment. A single newly emerged, healthy female was then introduced with one pre-parasitized egg mass into a finger tube at room temperature (26 ± 1 °C). Using a stereomicroscope, the female’s behavior was monitored in real time. Eggs upon which *T. remus* exhibited either oviposition-marking or probing behavior were specifically identified and marked. Following the completion of these behaviors, the marked eggs were incubated for one day before being dissected. The number of first-instar *T. remus* larvae present within each dissected host egg was observed and recorded. The experiment was conducted in five replicates, each utilizing a set of 30 dissected eggs.

#### 2.3.5. Self-Discrimination of *T. remus* Towards Marked Host Eggs Stored at Different Temperatures

Fresh *S. frugiperda* egg masses (30 eggs/mass) were exposed to individual, newly emerged *T. remus* females in finger tubes and allowed to parasitize completely under controlled conditions (26 ± 1 °C, 70 ± 5%RH, 14L:10D photoperiod, 1000 lux). Following parasitization, the *T. remus* individuals and their corresponding parasitized egg masses were separated. The egg masses were then stored for one hour at one of five designated temperatures (16, 21, 26, 31 or 36 °C) within climate chambers. Subsequently, each original *T. remus* was re-introduced to its respective egg mass in a finger tube. The re-marking behavior exhibited by *T. remus* towards the previously parasitized eggs was observed under a stereomicroscope at room temperature for a duration of 10 min, during which the average number of re-marked eggs was recorded. Five replicates were performed per temperature.

#### 2.3.6. Conspecific Discrimination of *T. remus* Towards Marked Host Eggs Stored at Different Temperatures

Following the same procedure for parasitization and temperature treatment as in Section 2.3.5, the stored egg masses were then exposed to one of two types of naive conspecific females in finger tubes: (1) newly emerged females with no prior oviposition experience; (2) newly emerged females with prior oviposition experience. The re-marking behavior towards the parasitized eggs was observed for a duration of 10 min under a stereomicroscope at room temperature, and the average number of re-marked eggs was recorded and calculated. Each female type and temperature condition was tested in five replicates.

#### 2.3.7. Self-Discrimination of *T. remus* Towards Marked Host Eggs After Different Time Intervals

Fresh egg masses laid by the same *S. frugiperda* female on the same day were divided into smaller units of 25 eggs each. Each unit (small egg mass) was placed into a finger tube with a single newly emerged *T. remus* female (1:1 ratio) and allowed to be fully parasitized. Following parasitization, the female wasp and the egg mass were separated. The parasitized eggs were then incubated in a climate chamber (26 ± 1 °C, 70 ± 5% RH, 14L:10D photoperiod, 1000 lux) for different time intervals (0, 1, 2, 4, 6, 10 or 12 h). Subsequently, each original wasp was then re-introduced to its respective time-interval egg mass. The re-marking behavior was observed for a period of 10 min at room temperature using a stereomicroscope, during which the average number of re-marked eggs was recorded and calculated. Five replicates were performed for each time interval.

#### 2.3.8. Conspecific Discrimination of *T. remus* Towards Marked Host Eggs After Different Time Intervals

Using the same parasitization and time-interval storage procedure as in Section 2.3.7, the stored egg masses were subsequently exposed to one of two types of naive conspecific females: (1) newly emerged females with no prior oviposition experience; (2) newly emerged females with prior oviposition experience. The re-marking behavior of *T. remus* on parasitized *S. frugiperda* egg masses was observed using a stereomicroscope at room temperature for a period of 10 min. The number of eggs that were re-marked was recorded and the parasitism rate was calculated. This experiment was replicated five times for each combination of female type and storage time interval.

#### 2.3.9. Data Analysis

Statistical analyses were conducted using SPSS 27.0. One-way analysis of Variance (ANOVA) was performed, and multiple comparison tests, including Duncan’s multiple range test, Tukey’s honestly significant difference (HSD) test, Games–Howell’s post hoc test, Dunnett’s test, and Dunnett’s T3 post hoc test, were applied according to the data characteristics (e.g., variance homogeneity and comparison purpose) to assess significant differences in the following datasets: parasitism efficiency of a single female on host eggs at different densities within 0.5 h, maximum parasitism rate across different exposure times, re-marking behavior on host eggs, and host discrimination (both self and conspecific) under different temperatures and time intervals. Data recording was performed using Microsoft Excel 19. (Microsoft Office, Redmond, WA, USA) and GraphPad Prism 9.5 was used for graph plotting (GraphPad Software, Inc., San Diego, CA, USA).

## 3. Results

### 3.1. Parasitization Behavior of T. remus

**Figure 1:**
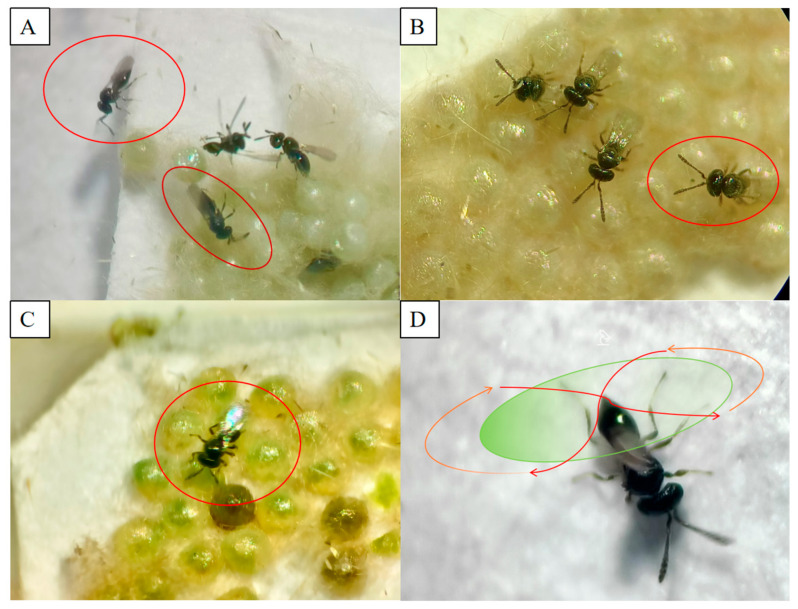
Stages of parasitism by *T. remus* on *S. frugiperda* eggs. (**A**) Searching and inspection; (**B**) probing and oviposition; (**C**) oviposition marking; (**D**) trail marking.

The oviposition behavior of *T. remus* encompasses a series of discrete actions, including searching, probing, oviposition, marking, and ovipositor grooming (Figure 1). During the searching phase, the female employs her antennae to continuously tap the surroundings of the host egg mass. Upon locating a host egg mass, she immediately climbs onto it and repeatedly taps each egg with her antennae for assessment. Eggs deemed unsuitable are abandoned in favor of further inspection. When an egg is considered appropriate, the female circles it or pauses to identify an optimal oviposition site using her ovipositor (Figure 1A). In the oviposition stage, if the egg proves unsuitable, the female retracts her ovipositor within a few seconds and resumes searching for other suitable eggs. Conversely, if the egg is suitable, she supports her body with her hind legs, inserts her ovipositor into the host egg, and deposits an egg internally (Figure 1B). Following oviposition, the ovipositor is withdrawn, and the female performs a distinctive “8”-shaped movement with the ovipositor tip on the egg surface to mark the parasitized egg, subsequently grooming the ovipositor (Figure 1C). This characteristic “8” marking serves as a reliable indicator of successful parasitization (Figure 1D). Additionally, it was observed that *S. frugiperda* egg masses are frequently covered by scales. The female detects interstitial gaps between these scales using her antennae to access the eggs, and may actively remove or displace portions of the scale layer to reach unparasitized eggs prior to probing and ovipositing.

Moreover, *T. remus* demonstrates the ability to recognize parasitized eggs during the searching phase. Upon encountering an egg that has been previously parasitized or marked, the female promptly avoids or bypasses it without extended inspection. When antennal tapping alone does not provide sufficient information to ascertain whether the egg has been parasitized or marked, she engages in ovipositor probing to assess the internal state of the egg. If the internal conditions are deemed unsuitable, such as the presence of prior parasitism, she retracts her ovipositor and departs. Conversely, if the egg is suitable, she proceeds to oviposit and subsequently deposits an oviposition mark on the egg’s surface.

### 3.2. Parasitism Efficiency of an Individual T. remus Female on Host Eggs at Different Densities

**Figure 2:**
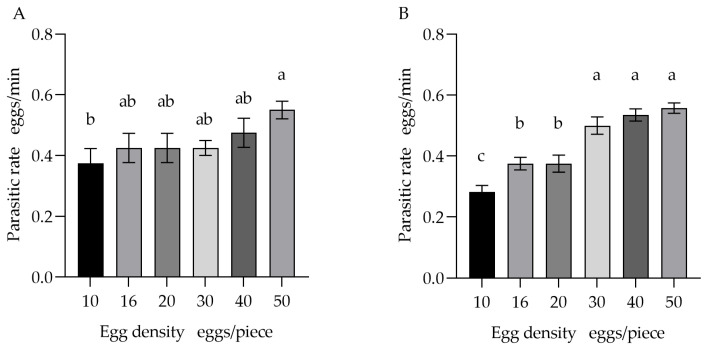
Parasitism efficiency of a single *T. remus* female on host eggs at different densities over two observation periods: (**A**) 10 min and (**B**) 30 min. Note: Data in the same row were analyzed by one-way ANOVA; different lowercase letters indicate significant differences at the level of *p* < 0.05 according to Duncan’s multiple range test.

As shown in Figure 2, the parasitism rate of a single *T. remus* female gradually increased with host egg density and eventually plateaued. Within 10 min, parasitism efficiency was significantly higher (*F* = 2.035, *p* < 0.05) for the 50-egg masses compared to the 10-egg masses. No other significant differences (*F* = 2.035, *p* > 0.05) were detected among the other density groups (16, 20, 30, 40 eggs) relative to these two extremes within this period.

Within 30 min, parasitism efficiency at the lowest density (10 eggs) was significantly lower than at densities of 16 and 20 eggs (*F* = 22.575, *p* < 0.05), and markedly lower than at densities of 30, 40, and 50 eggs (*p* < 0.01). Furthermore, the rates for the 16- and 20-egg masses were also significantly lower than those for the 30-, 40-, and 50-egg masses (*F* = 22.575, *p* < 0.05). These results indicate that as host density increases, the time required by a single female for host searching significantly decreases, eventually stabilizing at higher densities.

### 3.3. Parasitism Rate of an Individual T. remus Female in Relation to Host Exposure Time

**Figure 3:**
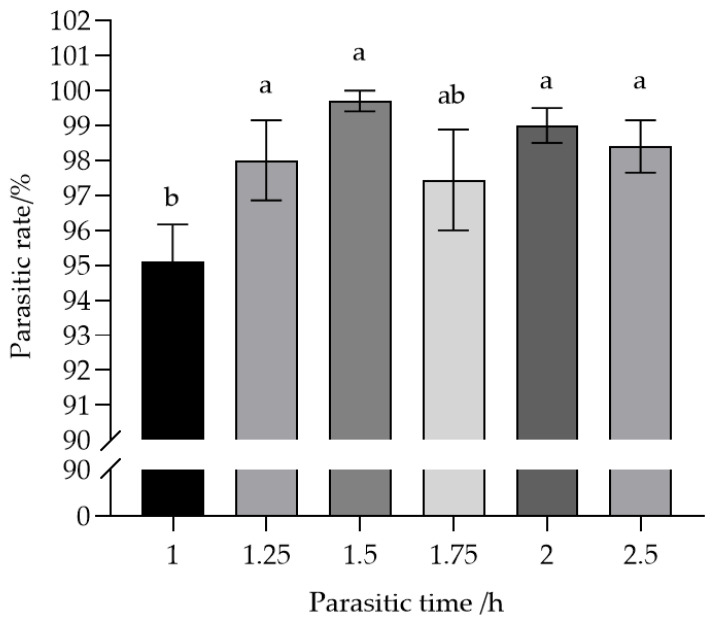
Parasitism rates exhibited by individual female *T. remus* on host egg masses at different time intervals. Note: Data in the same row were analyzed by one-way ANOVA; different lowercase letters indicate significant differences at the level of *p* < 0.05 according to Games–Howell’s post hoc test.

As shown in Figure 3, the parasitism rate achieved by a single *T. remus* female increased gradually as the exposure time extended up to 1.5 h, then slightly decreased and eventually stabilized. Except for the comparison with 1.75 h (*F* = 2.93, *p* > 0.05), the parasitism rate at 1 h showed significant differences (*F* = 2.93, *p* < 0.05) from those at 1.25 h, 1.5 h, 2 h, and 2.5 h. The highest parasitism rate (99.7%) was observed at 1.5 h of exposure, while the lowest rate (95.0%) occurred at 1 h. These results indicate that a parasitism duration of 1.5 h is optimal for a single female *T. remus* to achieve the highest parasitism rate on a 30-egg mass of *S. frugiperda*.

### 3.4. Parasitism Recognition of T. remus Towards Parasitized Host Eggs

*T. remus* exhibits marking behavior following oviposition on *S. frugiperda* egg masses, coupled with the capacity to recognize these marks. In the present study, the term “initial marking” denotes the mark deposited immediately after oviposition on fresh, unparasitized eggs. “Re-marking” refers to the marking applied subsequent to a second oviposition event on eggs that have already been parasitized. The term “re-probing” describes the behavior wherein the ovipositor is reinserted into a parasitized egg and then withdrawn without leaving a mark.

Oviposition was consistently accompanied by egg surface marking (Figure 1C,D). Dissection analyses revealed that eggs with initial marks contained no more than one larva, with an initial parasitism rate of 85.4%. Among eggs with re-marks, the distribution was as follows: 23 eggs contained two larvae, 5.8 eggs contained one larva, and 1.4 eggs contained no larvae. When parasitism rates were calculated based on the number of eggs containing two larvae, the rate was 76.6%. Statistical analysis indicated no significant difference (*F* = 1.417, *p* > 0.05) between the parasitism rates associated with initial marking and re-marking. Furthermore, no host egg was found to contain more than two larvae, indicating that each marking event corresponds to the deposition of a single egg within the host (Table 2).

Additionally, re-probing behavior was observed. Dissection of the eggs subjected to this behavior revealed that no egg contained two larvae. Among these eggs, 23.8 eggs contained one larva and 6.2 eggs contained none, with a resulting parasitism rate of 79.2%. Statistical analysis revealed no significant differences (*F* = 1.417, *p* > 0.05) in parasitism rates among initially marked, re-marked, and re-probed eggs. These findings indicate that re-probing does not result in oviposition and represents an additional form of host discrimination during oviposition.

### 3.5. Host Recognition by T. remus of Self-Marked Eggs Stored at Different Temperatures

**Figure 4:**
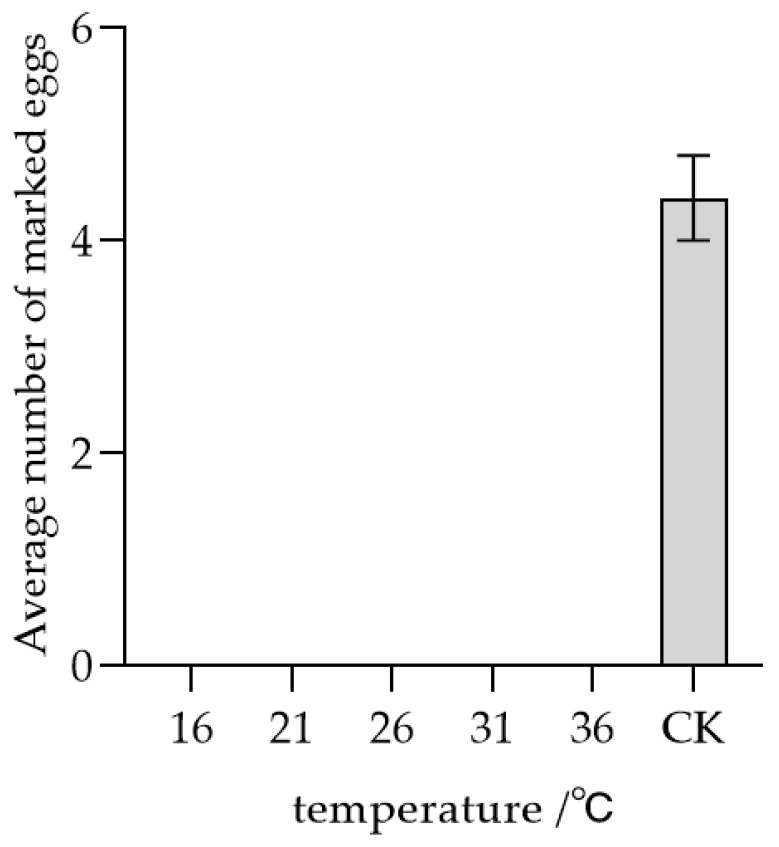
Parasitic recognition of *T. remus* self in relation to marked host eggs stored at different temperature conditions. Note: CK: Mean number of fresh host eggs marked by newly emerged *T. remus* females within 10 min at 26 °C. Hereafter the same.

As illustrated in Figure 4, the storage temperature of host eggs significantly influenced the parasitism recognition behavior exhibited by female *T. remus*. At temperatures of 16, 21, 26, 31, and 36 °C, the mean number of eggs marked during secondary parasitism recognition on treated host-marked eggs was 0, whereas in the CK group (first oviposition marking at 26 °C within 10 min), the mean number of eggs marked was 4.4. These results indicate that self-marked eggs stored between 16 °C and 36 °C consistently elicited host recognition behavior, effectively preventing re-parasitism by *T. remus*.

### 3.6. Host Recognition by Conspecific T. remus Females of Marked Eggs Stored at Different Temperatures

**Figure 5:**
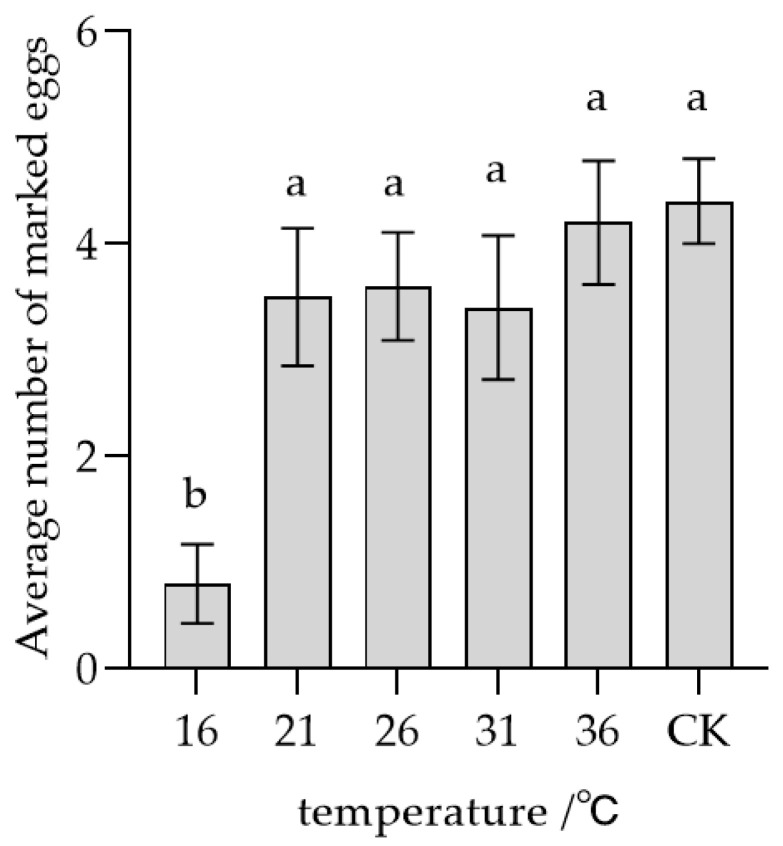
Parasitic recognition of female *T. remus* with no oviposition experience on marked host eggs stored at different temperature conditions. Note: Data in the same row were analyzed by one-way ANOVA; different lowercase letters indicate significant differences at the level of *p* < 0.05 according to the Dunnett’s and Tukey’s HSD post hoc tests.

The average number of eggs marked by conspecific *T. remus* females with no prior oviposition experience increased as the storage temperature of the eggs rose (Figure 5). Specifically, the mean quantities of marked eggs after one hour of storage at 16, 21, 26, 31, and 36 °C were 0.8, 3.5, 3.6, 3.4, and 4.2 eggs, respectively. Statistical analysis revealed a significant difference (*F* = 6.001, *p* < 0.05) between eggs stored at 16 °C and those stored at temperatures ranging from 21 to 36 °C, as well as the control group (CK), which consisted of eggs marked at 26 °C within 10 min. However, no significant differences (*F* = 6.001, *p* > 0.05) were detected among eggs stored at temperatures ranging from 21 to 36 °C or the control. These results indicate that only eggs stored at 16 °C for one hour were effectively recognized by conspecific unmated females, suggesting that the marking pheromone exhibited reduced volatilization at this lower temperature, thereby facilitating recognition.

**Figure 6:**
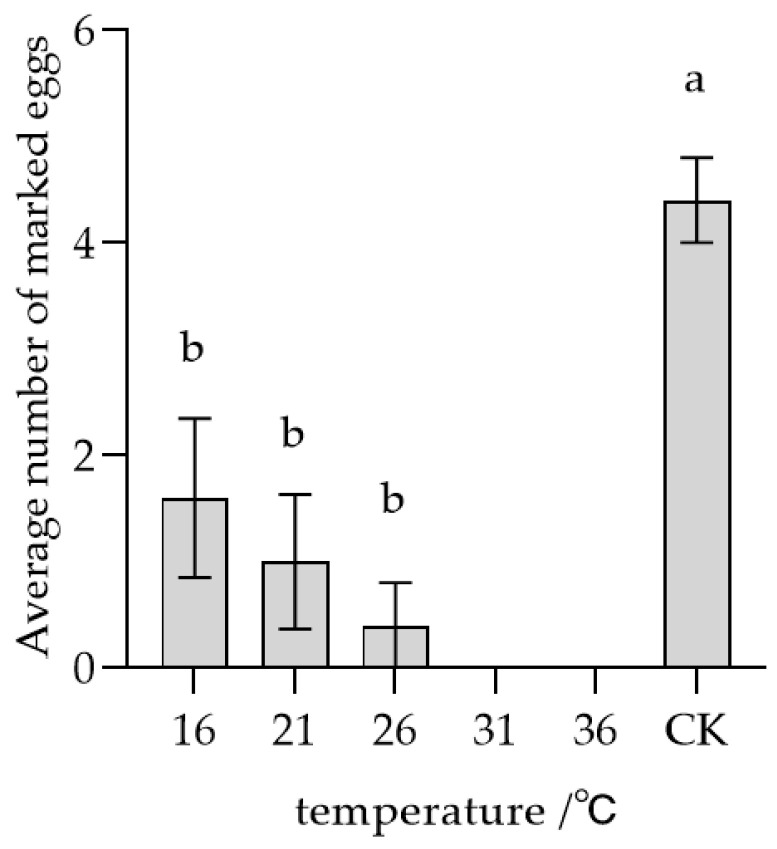
Parasitic recognition of female *T. remus* with prior oviposition experience on marked host eggs stored at different temperature conditions. Note: Data in the same row were analyzed by one-way ANOVA; different lowercase letters indicate significant differences at the level of *p* < 0.05 according to Dunnett’s T3 post hoc test.

Conversely, the mean quantity of eggs marked by conspecific females with prior oviposition experience exhibited a decline as temperature increased (Figure 6). The average numbers of marked eggs stored at 16, 21, 26, 31, and 36 °C were 1.6, 1.0, 0.4, 0, and 0 grains, respectively. Each treatment group showed a statistically significant difference (*F* = 13.081, *p* < 0.05) compared to the control (CK), although no significant differences were detected among the temperature treatments themselves. These findings imply that experienced females were able to recognize marked eggs across all storage temperatures, with recognition intensity increasing with temperature, indicating that their response may be influenced by pheromone concentration.

### 3.7. Host Recognition of Self-Marked Eggs by T. remus over Different Time Intervals

**Figure 7:**
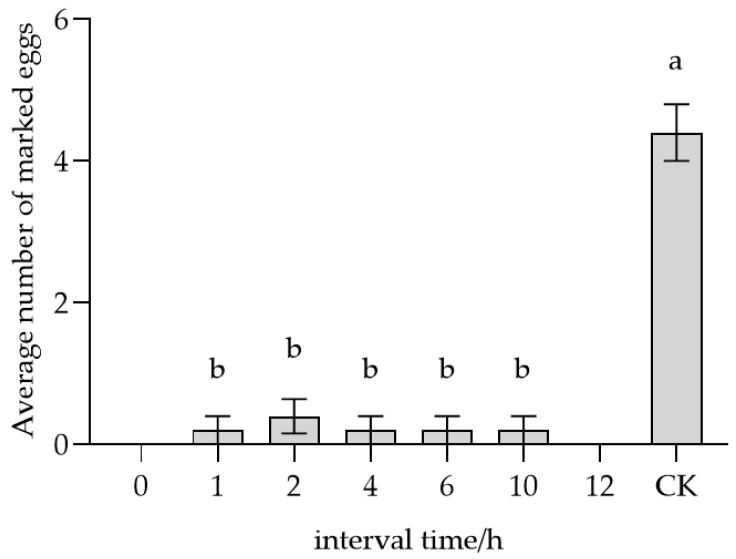
Parasitic recognition of self-parasitized host eggs at different time intervals by *T. remus.* Note: Data in the same row were analyzed by one-way ANOVA; different lowercase letters indicate significant differences at the level of *p* < 0.05 according to Dunnett’s T3 post hoc test.

*T. remus* females exhibited the ability to recognize their own marked eggs over various time periods (Figure 7). The average number of eggs remarked at 0, 1, 2, 4, 6, 10, and 12 h were 0, 0.2, 0.4, 0.2, 0.2, 0.2, and 0 grains, respectively. Statistical analysis revealed that all time intervals from 1 to 10 h showed significant differences (*F* = 47.398, *p* < 0.05) compared to the (CK),which had 4.4 eggs marked at 26 °C within 10 min. These findings indicate that self-marked eggs were consistently recognized by *T. remus* females over the examined time.

### 3.8. Host Recognition by Conspecific T. remus on Marked Eggs over Different Time Intervals

**Figure 8:**
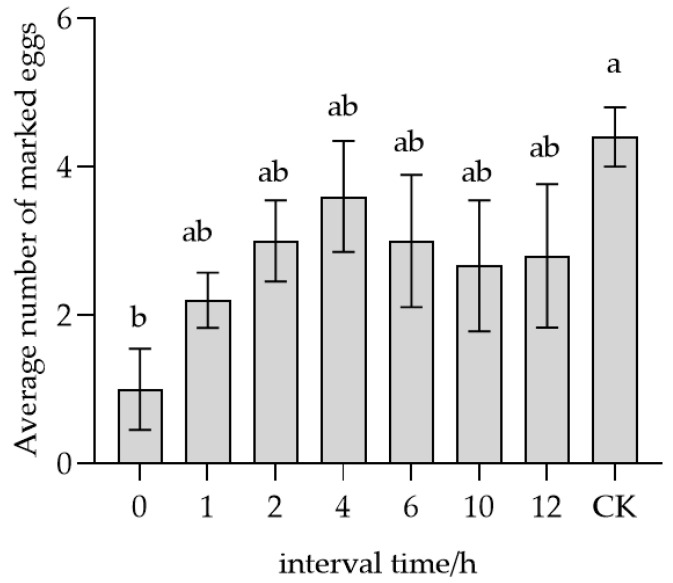
Parasitic recognition of female *T. remus* without oviposition experience on marked host eggs stored at different intervals. Note: Data in the same row were analyzed by one-way ANOVA; different lowercase letters indicate significant differences at the level of *p* < 0.05 according to Dunnett post hoc test.

As illustrated in Figure 8, the average number of eggs marked by conspecific unmated females initially increased and then stabilized over time. At 0 h, the mean number of marked eggs (1 egg) was significantly lower (*F* = 2.146, *p* < 0.05) compared to the CK (4.4 eggs). The mean values recorded at 1, 2, 4, 6, 10, and 12 h were 2.2, 3.0, 3.6, 3.0, 2.7, and 2.8 eggs, respectively, with no statistically significant differences observed among these intervals. These findings indicate that the recognition ability of conspecific unmated females declined over time, likely due to natural degradation of the host-marking pheromone.

**Figure 9:**
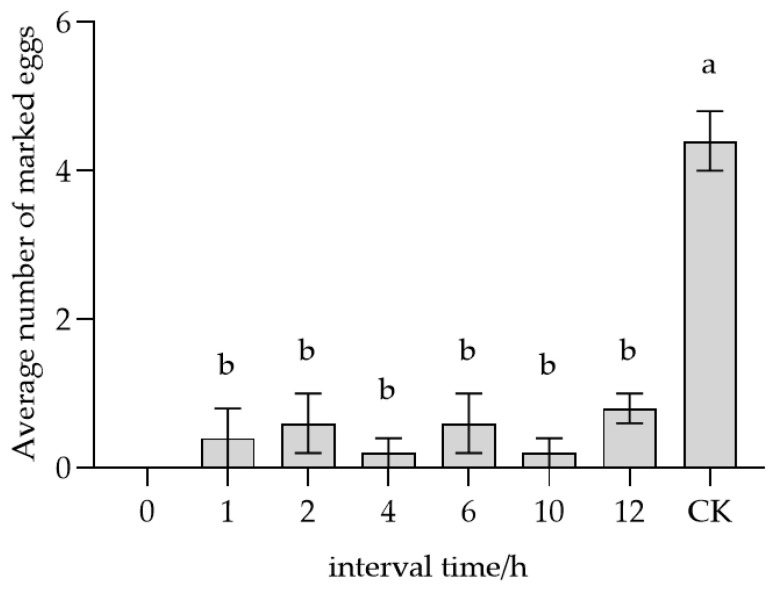
Parasitic recognition of female *T. remus* with oviposition experience on marked host eggs stored at different intervals. Note: Data in the same row were analyzed by one-way ANOVA; different lowercase letters indicate significant differences at the level of *p* < 0.05 according to Dunnett’s T3 post hoc test.

In contrast, experienced conspecific females consistently recognized marked eggs across all examined time intervals (Figure 9). Each time points showed a statistically significant difference (*F* = 21.774, *p* < 0.05) compared to the CK. The average quantities of marked eggs detected at 0, 1, 2, 4, 6, 10, and 12 h were 0, 0.4, 0.6, 0.2, 0.6, 0.2, and 0.8 grains, respectively, with no significant differences among intervals. These findings suggest that experienced females retained the ability to recognize marked eggs for up to 12 h, potentially attributable to learning experience associated with prior oviposition behavior.

## 4. Discussion

The parasitic behavior exhibited by numerous parasitoid wasps encompasses several stages, including host location, host examination, oviposition probing, and host-marking [40,41,42]. Among these behaviors, host-marking serves as an effective behavior to prevent superparasitism. Comparative analysis between the genera *Telenomus* and *Trissolcus* confirms that host recognition is widespread in both groups. Most *Trissolcus* species employ a uniform marking behavior: they drag the ovipositor in a characteristic “8”-shaped pattern over the egg surface to deposit the marking pheromone. Notably, *T. remus* in this study also employs this same “8”-shaped pattern marking behavior. Within the genus *Telenomus*, species such as *T. busseolae* Gahan and *T. isis* Polaszek, also perform similar abdominal twisting to trace an “8” pattern on the host egg [43], similar to *T. remus*. In contrast, some *Telenomus* species exhibit different marking motions, including continuous rubbing along a curved trajectory, lateral sweeping, or drawing irregular circular patterns around the oviposition site. Despite exhibiting greater diversity in marking behaviors than *Trissolcus,* most *Telenomus* species nonetheless share the key characteristic of host recognition with the latter. Recognition of these marking behaviors allows parasitoids to avoid intra- and interspecific competition, thereby reducing egg wastage and improving oviposition efficiency. This in turn stimulates parasitoids to search for more unparasitized hosts, which is highly advantageous for biological control programs.

Host density and the duration of parasitization are established factors influencing parasitism rates [44,45]. Research indicates that under conditions of host egg scarcity, *T. remus* exhibits protective behavior towards parasitized hosts, while prolonged parasitization can lead to superparasitism [3]. To evaluate how these factors affect *T. remus*, the present study confirms that both are critical in determining its parasitism rate. Short-term oviposition behavior (observed over 10 to 30 intervals) reflects the search efficiency of *T. remus.* Search time decreased with increasing host density until reaching a plateau, demonstrating a density-dependent “convenience effect”. These findings aligns with Morales-Sánchez et al., who reported that the parasitism rate of *T. remus* in response to host density follows Holling’s Type II functional response model [46]. A similar pattern has been documented in *Meteorus pulchricornis* (Wesmael), whose parasitism rate decreased with increasing host density below a threshold of about 8 hosts per plant, and stabilized near 50% beyond that threshold, indicating an upper limit to the parasitoid’s parasitic capacity [47]. Longer parasitization durations (e.g., 1.5 h) reflect the total parasitic capacity of *T. remus*. At a host density of 30 eggs per egg mass, a 1.5 h exposure achieved the maximum parasitism rate. Shorter exposure times were insufficient to achieve full parasitic potential, while excessively prolonged durations potentially diminished efficiency due to oviposition fatigue and excessive probing behavior. Consequently, a parasitization duration of 1.5 h was identified as optimal and was employed as the standard condition in subsequent experimental procedures.

In natural environments, many parasitoid species possess the ability to discriminate parasitized hosts [11], thereby avoiding oviposition in hosts that have already been parasitized and consequently minimizing intraspecific competition among their offspring [48,49,50]. Research indicates that *T. remus* exhibits a pronounced host discrimination ability using sensory organs such as antennae and ovipositor to detect parasitized hosts and prevent superparasitism. Behavioral studies corroborate that females typically lay only one egg per host and avoid re-parasitizing the same egg, aligning with observations reported by Xue et al. [3]. Despite this strong discriminatory behavior, the presence of multiple larvae within a single host egg has been observed both in natural and laboratory conditions [3]. However, usually only one wasp larva successfully reaches maturity [51]. This phenomenon may result from intraspecific larval competition or occasional failures in recognizing host-marking pheromones; further research is required to elucidate the underlying mechanisms.

Factors such as prior oviposition experience [52], temperature [53], and time intervals [43,54] can significantly influence parasitoid discrimination behavior. The present study demonstrated that experienced females of *T. remus* could recognize marked host eggs across a temperature range (16–36 °C) in both self- and conspecific recognition assays, whereas inexperienced females only discriminated hosts marked at 16 °C. These results suggest that lower temperatures may better preserve host-marking substances (e.g., pheromones or cuticular compounds), while higher temperatures (21–36 °C) likely accelerate their degradation or volatilization. Additionally, experienced females may also detect fainter chemical signals, possibly due to sensitized sensory perception. Moreover, experienced females maintained effective recognition of parasitized hosts for up to 12 h, whereas naïve females only recognized eggs marked at 0 h. This implies that experienced *T. remus* may rely on internal memory (e.g., long-term memory) to compensate for fading external chemical signals. Supporting evidence from Wang et al. [24] reported that *T. ostriniae* Pang et Chen with one or two oviposition experiences required less time for host searching and oviposition, and rarely needed to probe host eggs with their ovipositors for recognition, in contrast to naive wasps and control groups. Similarly, *Trichogramma evanescens Westwood*, initially exhibited no innate response to benzyl cyanide, an odor associated with mated *Pieris brassicae* (Lin.), but after a single oviposition experience, individuals learned to associate this cue with successful parasitism and formed a long-term memory [55]. These findings further support that oviposition experience prolongs the persistence of host discrimination memory. This behavioral difference may reflect an evolutionary strategy whereby experienced females optimize resource utilization and avoid superparasitism through accumulated experience, thereby enhancing offspring survival. Conversely, inexperienced females may rely predominantly on immediate signals to minimize energy expenditure.

Thus, oviposition experience emerges as a primary determinant influencing host discrimination in *T. remus* females, a characteristic similarly observed in other parasitoid wasps. For instance, oviposition experience significantly enhances the discrimination ability of *Trichogramma chilonis* Ishii [52]. Individuals with prior oviposition experience demonstrate enhanced discriminatory performance, suggesting that accumulated experience facilitates environmental adaptation and strengthens long-term recognition memory—a crucial advantage for parasitoids characterized by brief lifespans and limited reproductive opportunities [55]. These findings are consistent with the documented roles of learning and memory functions in parasitoids as reported by Lewis and Takasu [56] and Wang et al. [24]. Through experience-enhanced discrimination, *T. remus* optimizes resource allocation, reduces intraspecific offspring competition, and ultimately increases reproductive success. This behavioral adjustment represents a strategic mechanism to maximize offspring survival.

From the standpoint of biological control, these results offer a foundation for enhancing the deployment strategies of *T. remus*. Subsequent investigations may delve deeper into molecular mechanisms that underpin this discriminatory capacity, including associated gene expression patterns or neural changes.

## Figures and Tables

**Table 1:** Comparison of marking behaviors between *Trissolcus* and *Telenomus* genera.

Genus	Species	Marking (Yes/No)	Marking Method	References
*Telenomus*	*T. sphingis*	Yes	Rubbing along a continuous curved trajectory on the egg surface with the ovipositor.	Rabb, R.L.; et al. 1970 [28]
	*T. fariai*	Yes	Rubbing the egg surface with the ovipositor.	Escalante, G. and Rabinovich, J.E. 1979 [29]
	*T. heliothidis*	Yes	Moving left and right along the egg surface with the ovipositor.	Strand, M.R. and Vinson, S.B. 1983 [30]
	*T. reynoldsi*	Yes	Drawing irregular circles on the egg surface with the ovipositor.	Cave, R.D.; et al. 1987 [31]
	*T. busseolae*	Yes	Smearing “8”-shaped marking on egg surface with the ovipositor.	Bruce, A.Y.; et al. 2021 [32]
	*T. isis*	Yes	Smearing “8”-shaped marking on egg surface with the ovipositor.	Bruce, A.Y.; et al. 2021 [32]
	*T. triptus*	Yes	Scraping the egg surface with the ovipositor.	Higuchi, H.and Suzuki, Y. 1996 [33]
	*T. euproctidis*	Yes	Making 1–3 lateral sweeps across the egg surface with the ovipositor.	Ou, X.; et al. 1996 [34]
	*T. podisi*	Yes	Not described.	Okuda, M.S. and Yeargan, K.V. 1988 [22]
	*T. dendrolimusi*	Yes	Not described.	Huang, Y.P.; et al. 1993 [8]
	*T. gifuensis*	Yes	Not described.	Mahmoud, A.M.A. and Lim, U.T. 2008 [35]
	*T. theophilae*	Yes	Not described.	Sun, S. 2003 [36]
*Trissolcus*	*T. nigripedius*	Yes	Smearing “8”-shaped marking on egg surface with the ovipositor.	Mahmoud, A.M.A. and Lim, U.T. 2007 [37]
	*T. japonicus*	Yes	Smearing “8”-shaped marking on egg surface with the ovipositor.	Li, Z. 2002 [38]
	*T. basalis*	Yes	Smearing “8”-shaped marking on egg surface with the ovipositor.	Weber, C.A. et al. 1996 [39]
	*T. utahensis*	Yes	Smearing “8”-shaped marking on egg surface with the ovipositor.	Weber, C.A. et al. 1996 [39]
	*T. euschisti*	Yes	Not described.	Okuda, M.S. and Yeargan, K.V. 1988 [22]

**Table 2:** Secondary labeling and detection recognition of *T. remus* on marked host eggs.

Parasitic Mode	Sample Size	Number of Larvae			Parasitism Rate/%
	n	2	1	0	
First marking	30	0	25.6	4.4	85.4 ± 0.03 a
Secondary marking	30	23	5.8	1.4	76.6 ± 0.04 a
Secondary probing	30	0	23.8	6.2	79.2 ± 0.04 a

Note: Mean ± standard error; Data in the same row were analyzed by one-way ANOVA; different lowercase letters indicate significant differences at the level of *p* < 0.05 according to Tukey’s HSD post hoc test. n: sample size; 2: two first instar larvae of *T. remus*; 1: one first instar larva of *T. remus*; 0: 0 first instar larva of *T. remus*.

## Data Availability

The original contributions presented in this study are included in the article. Further inquiries can be directed to the corresponding author.

## References

[1] Prokopy R.J. (1981). Epideictic pheromones that influence spacing patterns of phytophagous insects. Semiochemicals: Their Role in Pest Control.

[2] Salt G. Competition among insect parasitoids. Mechanisms in biological competition. Proceedings of the Symposium of the Society for Experimental Biology.

[3] Xue T., Pan J., LIU L., Xu T., Huang J., He Y., Wang Z. (2024). Intraspecific host discrimination and superparasitism in *Telenomus remus* (Nixon), an egg parasitoid of *Spodoptera frugiperda* (J. E. Smith). Chin. J. Biol. Control.

[4] Hardy I.C.W., Briffa M. (2013). Introduction to Animal Contests.

[5] Zhu X., Liu Q., Xu C.Q., Guo K., Xu R., Qiao H.L., Chen J. (2021). Advances in research on sensilla structure andfunction in parasitic wasps. Chin. J. Appl. Entomol..

[6] Eisenstein E., Reep R. (1985). Behavioral and cellular studies of learning and memory in insects. Compr. Insect Physiol. Biochem. Pharmacol..

[7] Nufio C.R., Papaj D.R. (2001). Host marking behavior in phytophagous insect and parasitoids. Entomol. Exp. Appl..

[8] Huang Y.P., Wang S.F., Tang D.W., Ren L.Z. (1993). Host discrimination and marking pheromone of *Telenomus dendrolimusi*. J. Cent. South Univ. For. Technol..

[9] Li G., Mu L. (2006). Competition among parasitoids for host during foraging and oviposition. Acta Ecol. Sin..

[10] Wang H., Cheng M., Wang S., Wen Y., Cui J., Li J. (2021). Discrimination against already parasitized hosts by *Aprostocetus prolixus* (Hymenoptera: *Eulophidae*). Acta Sericologica Sin..

[11] Li G. (2006). Host-marking in hymenopterous parasitoids. Acta Entomol. Sin..

[12] Beli W.J. (1985). Comprehensive insect physiology, biochemistry and pharmacology: Edited by G. A. Kerkut and L. I. Gilbert. Pergamon press. Oxford. 1985. Volume 9. Behaviour. 734 + xvi pp. Int. J. Biochem..

[13] Klomp H., Teerink B., Ma W.C. (1980). Discrimination between parasitized and unparasitized hosts in the egg parasite *Trichogramma embryophagum* (Hym.: *Trichogrammatidae*): A matter of learning and forgetting. Neth. J. Zool..

[14] Van Alphen J., Nell H. (1981). Superparasitism and host discrimination by *Asobara Tabida* Nees (Braconidae: *Alysiinae*), a Larval parasitoid of drosophilidae. Neth. J. Zool..

[15] Ikawa T., Suzuki Y. (1982). Ovipositional experience of the gregarious parasitoid, *Apanteles glomeratus* (Hymenoptera: *Braconidae*), influencing her discrimination of the host larvae, *Pieris rapae crucivora*. Appl. Entomol. Zool..

[16] Wylie H. (1965). Some factors that reduce the reproductive rate of *Nasonia vitripennis* (Walk.) at high adult population densities. Can. Entomol..

[17] Chen X., Bordini G.P., Stansly P.A. (2016). Avoidance of parasitized hosts by female wasps of *Tamarixia radiata* (Hymenoptera: *Eulophidae*), Parasitoid of *Diaphorina citri* (Hemiptera: *Liviidae*), vector of citrus greening disease. Fla. Entomol..

[18] Mehrnejad M.R., Copland M.J.W. (2007). Host discrimination by the endoparasitoid *Psyllaephagus pistaciae* (Hymenoptera: *Encyrtidae*): A case of time-dependent ability. Biocontrol Sci. Technol..

[19] Li X., Li B., Meng L. (2018). Oviposition strategy for superparasitism in the gregarious parasitoid *Oomyzus sokolowskii* (Hymenoptera: *Eulophidae*). Bull. Entomol. Res..

[20] Field S.A., Keller M.A. (1999). Short-term host discrimination in the parasitoid wasp *Trissolcus basalis* Wollaston (Hymenoptera: *Scelionidae*). Aust. J. Zool..

[21] Oudenhove L.V., Billoir E., Boulay R., Bernstein C., Cerda X. (2011). Temperature limits trail following behaviour through pheromone decay in ants. Naturwissenschaften.

[22] Okuda M.S., Yeargan K.V. (1988). Intra- and interspecific host discrimination in *Telenomus podisi* and *Trissolcus euschisti* (Hymenoptera: *Scelionidae*). Ann. Entomol. Soc. Am..

[23] McKay T., Broce A.B. (2004). Discrimination of Self-parasitized hosts by the pupal parasitoid *Muscidifurax zaraptor* (Hymenoptera: *Pteromalidae*). Ann. Entomol. Soc. Am..

[24] Wang S.Q., Lian Y.G., Kang Z.J., Mo T.L. (2007). Effects of host marking pheromones and experiences on oviposition behavior of *Trichogramma ostriniae*. Acta Ecol. Sin..

[25] Wang J.W., Zhou Q., Xu T., Luo S.M. (2003). Roles of volatile infochemicals and learning behavior in the host selection process of *Anastatus japonicus*. Acta Ecol. Sin..

[26] Liu S.M. (2023). The Strategy and Mechanism of Avoiding Superparasitism of *Asobara japonica*. Master’s Thesis.

[27] Wei K. (2016). Behavioral and Developmental Strategies for Adapting the Heterogeneous Environmental Conditions of the Generalist Ectoparasitoid *Sclerodermus pupariae* (Hymenoptera: *Bethylidae*). Ph.D. Thesis.

[28] Rabb R.L., Bradley J.R. (1970). Marking host eggs by *Telenomus sphingis*. Ann. Entomol. Soc. Am..

[29] Escalante G., Rabinovich J.E. (1979). Population dynamics of *Telenomus fariai* (Hymenoptera: *Scelionidae*), a parasite of Chagas’ disease vectors IX. Larval competition and population size regulation under laboratory conditions. Res. Popul. Ecol..

[30] Strand M.R., Vinson S.B. (1983). Host acceptance behavior of *Telenomus heliothidis* (Hymenoptera: *Scelionidae*) toward *Heliothis virescens* (Lepidoptera: *Noctuidae*). Ann. Entomol. Soc. Am..

[31] Cave R.D., Gaylor M.J., Bradley J.T. (1987). Host handling and recognition by *Telenomus reynoldsi* (Hymenoptera: *Scelionidae*), an egg parasitoid of *Geocoris* spp. (Heteroptera: *Lygaeidae*). Ann. Entomol. Soc. Am..

[32] Bruce A.Y., Schulthess F., Makatiani J.K., Tonnang H.E.Z. (2021). Oviposition behavior of *Telenomus busseolae*, *Telenomus isis* and *Trichogramma bournieri* on eggs of east African cereal stemborers. Int. J. Trop. Insect Sci..

[33] Higuchi H., Suzuki Y. (1996). Host handling behavior of the egg parasitoid *Telenomus triptus* to the egg mass of the stink bug *Piezodorus hybneri*. Entomol. Exp. Appl..

[34] Ou X., Jiang H., Chen C. (1996). An Observation on parasitizing Be-havior of *Telenomus euproctidis* Wilcox (Hymenoptera: *Scelionidae*). J. Hunan Agric. Univ..

[35] Mahmoud A.M.A., Lim U.T. (2008). Host discrimination and interspecific competition of *Trissolcus nigripedius* and *Telenomus gifuensis* (Hymenoptera: *Scelionidae*), sympatric parasitoids of *Dolycoris baccarum* (Heteroptera: *Pentatomidae*). Biol. Control.

[36] Sun S. (2003). Study on the Transfer Pathway and Activate Peptide of Host Recognition Kairomone for *Telenomus theophilae*. Ph.D. Thesis.

[37] Mahmoud A.M.A., Lim U.T. (2007). Evaluation of cold-stored eggs of *Dolycoris baccarum* (Hemiptera: *Pentatomidae*) for parasitization by *Trissolcus nigripedius* (Hymenoptera: *Scelionidae*). Biol. Control.

[38] Li Z. (2002). Preliminary Studies on the *Trissolcus japonicus* Ashmead(Hymenoptera: *Scelionidae*), a Parasitoid of *Halyomorpha picus* Fabricius (Heteroptera: *Pentatomidae*) Eggs. Master’s Thesis.

[39] Weber C.A., Smilanick J.M., Ehler L.E., Zalom F.G. (1996). Ovipositional behavior and host discrimination in three scelionid egg parasitoids of Stink Bugs. Biol. Control.

[40] Tian Z., Wang Y., Sun T., Hu X., Hao W., Zhao T., Wang Y., Zhang L., Jiang X., Turlings T.C.J. (2025). An egg parasitoid assesses host egg quality from afar using oviposition-induced plant volatiles. Curr. Biol. CB.

[41] Libersat F., Delago A., Gal R. (2009). Manipulation of host behavior by parasitic insects and insect parasites. Annu. Rev. Entomol..

[42] Lv Y., Guo X., Qin W., Meng L., Li B. (2024). Effects of host life stage and female wasp’s age on parasitizing behaviors and offspring developmental consequences in *Aenasius arizonensis* (Hymenoptera: *Encyrtidae*). Chin. J. Appl. Entomol..

[43] Chabi-Olaye A., Schulthess F., Poehling H.-M., Borgemeister C. (2001). Host location and host discrimination behavior of *Telenomus isis*, an egg parasitoid of the African cereal stem borer *Sesamia calamistis*. J. Chem. Ecol..

[44] Wang X., Aparicio E.M., Duan J., Gould J., Hoelmer K.A. (2020). Optimizing parasitoid and host densities for efficient rearing of *Ontsira mellipes* (Hymenoptera: *Braconidae*) on Asian *Longhorned Beetle* (Coleoptera: *Cerambycidae*). Environ. Entomol..

[45] Power N.R. (2020). Evaluation of the Parasitoid *Ooencyrtus mirus* (Hymenoptera: Encyrtidae) as a Potential Biological Control Agent of *Bagrada hilaris* (Heteroptera: *Pentatomidae*). Ph.D. Thesis.

[46] Morales S.J., Gallardo V.J.S., Vásquez C., Ríos Y. (2001). Functional response of *Telenomus remus* (Hymenoptera: *Scelionidae*) to the fall army worm eggs. J. Bioagro..

[47] Zhang B., Meng L., Li B.P. (2012). Oviposition selection behavior of *Meteorus pulchricornis* (Hymenoptera: *Braconidae*) in the field. J. Nanjing Agric. Univ..

[48] Hanan A., Shakeel M., He X.Z., Razzaq A., Wang Q. (2016). Superparasitism and host discrimination behavior of *Eretmocerus warrae* Naumann & Schmidt (Hymenoptera: *Aphelinidae*). Turk. J. Agric. For..

[49] Liang Q., Jia Y., Liu T. (2017). Self-and conspecific discrimination between unparasitized and parasitized green peach aphid (Hemiptera: Aphididae), by *Aphelinus asychis* (Hymenoptera: *Aphelinidae*). J. Econ. Entomol..

[50] Chen W.-b., Vasseur L., Zhang S.-q., Zhang H.-f., Mao J., Liu T.-s., Zhou X.-y., Wang X., Zhang J., You M.-s. (2020). Mechanism and consequences for avoidance of superparasitism in the solitary parasitoid *Cotesia vestalis*. Sci. Rep..

[51] Tang Y., Chen K., Xu Z. (2010). Study on ontogenesis of *Telenomus remus* Nixon (Hymenoptera: *Scelionidae*). J. Chang. Veg..

[52] Miura K., Matsuda S., Kobayashi M. (1994). Discrimination between parasitized and unparasitized hosts in an egg parasitoid, *Trichogramma chilonis* Ishii (Hymenoptera: *Trichogrammatidae*). Appl. Entomol. Zool..

[53] Alborn H.T., Lewis W.J., Tumlinson J.H. (1995). Host-specific recognition kairomone for the parasitoid *Microplitis croceipes* (*Cresson*). J. Chem. Ecol..

[54] Hougardy E., Hogg B.N. (2021). Host patch use and potential competitive interactions between two egg parasitoids from the family Scelionidae, candidate biological control agents of *Bagrada hilaris* (Hemiptera: *Pentatomidae*). J. Econ. Entomol..

[55] Huigens M.E., Pashalidou F.G., Qian M.-H., Bukovinszky T., Smid H.M., van Loon J.J., Dicke M., Fatouros N.E. (2009). Hitch-hiking parasitic wasp learns to exploit butterfly antiaphrodisiac. Proc. Natl. Acad. Sci. USA.

[56] Lewis W., Takasu K. (1990). Use of learned odours by a parasitic wasp in accordance with host and food needs. Nature.

